# Assessment of Nutritional Status Among Adolescent Boys in an Urban Population of South India

**DOI:** 10.5539/gjhs.v7n3p335

**Published:** 2015-03-10

**Authors:** Shahla Shafiee, Mohsen Mesgarani, Khyrunnisa Begum

**Affiliations:** 1 Childern and adolescents health research Center, Zahedan University of Medical Science, Zahedan, Iran; 2 Zahedan Health Promotion Research Center, Zahedan University of Medical Science, Zahedan, Iran; 3 Department of Studies in Food Science and Nutrition Manasagangotri University of Mysore, India

**Keywords:** adolescence, boys, nutrients, intake, deficiency

## Abstract

**Background:**

Deficiency of calories and certain micronutrients is known to cause growth faltering in children and adolescents. It is recognized that varieties of foods need to be consumed in order to meet requirements for essential nutrients. Lack of diversity in the diets is a serious problem among poor populations in the developing world. The extent of variations in intake of nutrients occurring in a homogeneous population provides useful information.

**Subjects and Methods:**

This study investigates the mean intake of nutrient by 1083 adolescent males, age 10-19 years, in comparison to the RDA values suggested by ICMR for Indians. Food intakes, social class and knowledge about health education were obtained by questionnaires. Descriptive statistics, non-parametric statistics, and Chi-Square tests were performed to and interpret the data, particularly hypothesis testing.

**Results:**

Mean intake of calories varied from 1512±532 for pre-adolescent to 1742±660 for post-adolescence, the differences in intake between pre-adolescence to adolescence was statistically significant. The intake was largely different compared to the respective RDAs including proteins which were markedly lower than the RDA. The mean intake increased linearly with the advancing stages of adolescence. Intake of calcium by boys during pre-adolescence and adolescence stage were lower by 20-30% as compared to the RDA, whereas the post-adolescent boys were found to consume a fair amount and met their RDAs. Intakes of iron and β-carotene were highly variable, the majority of the selected boys consumed much less than the RDAs. The differences in the intakes were statistically not significant.

**Conclusion:**

Mean intakes of nutrients indicate that the majority of the selected boys consumed protein, calories, iron, calcium and β carotene in three stages of adolescent markedly lower than the respective RDAs. Family type, birth order and SES correlated with nutrient intake among selected adolescent boys.

## 1. Introduction

Adolescence is a period of rapid growth and maturation in human development (Maiti et al., 2011). There has been a worldwide significant change in the nutritional status of adolescents during the past 2 decades because of global economic development and urbanization (Wang, Chen, Shaikh, & Mathur, 2009). In developing nations, deficiency of calories and certain micronutrients is known to cause growth faltering in children (Best et al., 2010; Srihari, Eilander, Muthayya, Kurpad, & Seshadri, 2007; World Health Organization, 2011). In this period intake of recommended quantities of nutrients is essential to express growth potential (Hardy et al., 2006; Sjöberg, Hallberg, Höglund, & Hulthén, 2003). One crucial component of the healthy development of adolescents is good nutrition, which influences adolescent health, learning, physical fitness, and ability to withstand stress, and work at maximum productivity (The world bank, 2004). There is evidence that nutritional and environment determinants of growth and development can influence health and wellbeing which in turn can influence nations’ burden of malnourished individuals and public health problems (Bree, Eaves, Dwyer, & van den Bree, 1999; Duyar & Özener, 2005; Neumark-Sztainer, Wall, Story, & Fulkerson, 2004).

Potentially, the inclusion of adolescent boys in nutrition and healthy lifestyle programs will contribute to the improved nutrition and health of families including men and women (Tiedemann & DasGupta, 2000). In studies about growth and development of children, it is conventional practice to measure usual intakes of nutrients and compare to the recommended allowances (Chitra & Reddy, 2007).

It is recognized that varieties of foods need to be consumed in order to meet requirements for essential nutrients. Lack of diversity in diets is particularly a serious problem among poor populations in the developing world, where diets are based predominantly on starchy staples and often include only seasonal fruits and vegetables and few or no animal products (Guidetti & Cavazza, 2008). Vegetarian diets can also provide adequate nutrients and energy to support growth and development if well planned; Vegan diets may lack calcium, iron, vitamins D, B-12 and other micronutrients (“Position of the American Dietetic Association: Individual-, Family-, School-, and Community-Based Interventions for Pediatric Overweight”, 2006).

This study examines the nutritional status of the adolescent boys in comparison to the RDA values suggested by ICMR for Indians (Indian Council of Medical Research, Expert group, 2009).

## 2. Material and Method

Mysore city is an urban area of Karnataka, South India, with the population of 0.710 million residing in several streets, according to the municipal corporation record based on the 2001 census. The survey was conducted in 2008-2009, when the population should be greater (Approximately 0.861 million). Approximately 20% of the population was adolescents with a male: female ratio of 1. Mysore city had a total of 55 schools, approximately half of them private and the rest government schools. These schools offer education from 6^th^ to 12^th^ standard for children aged 10 to 18 years. A cross sectional study was carried out in schools offering primary and higher primary education (6^th^ to 10^th^ standard) and pre-university colleges offering 11^th^ and 12^th^ standard courses. All schools were randomly selected based on random numbers, so as to include one each of government and a reputed private institute. We selected 2 primary schools, 2 high schools and 3 pre-university colleges for this study.

Using a random number generator a minimum of 120 boys from each age groups of 10-18 years (6^th^ to 12^th^ standard in schools) were selected from each school. Of a total 1220 subjects contacted, 137 were excluded due to various reasons and the rest 1083 volunteers were included in this study. Exclusion criteria were: lack of interest by the student (n= 22), no reply to the consent letter from parents (n=55), less than 10 year or above the 19 years of age based on their birthday (n=48), and presence of major illness or physical deformity (n=12).

All the boys and their parents were informed about the purpose and protocols of the study. A written informed consent was obtained from the parents of the wards; children whose parents agreed for their participation were included for the study. Approval for the study was granted by Human Ethics Committee, University of Mysore.

A self reporting questionnaire was developed using the English language for obtaining family data relating to socioeconomic status and personal information; the questionnaire was translated into the regional language (Kannada- a Dravidian language of south India) with the help of a language expert. The questionnaires were provided to boys depending on the language with which they felt comfortable to answer.

Lack of knowledge about health education was considered in relation to absence of knowledge about harmful effects of tobacco, alcoholism and Western type foods and the beneficial effects of exercise and prudent foods (fruits, vegetables, nuts and fish). The number of health educated subjects among those who were educated more than 10^th^ class was was also obtained by a questionnaire.

### 2.1 Social Class

A socio economic scale was developed for this study taking into account parent’s educational level, occupation, per capita income, type of house, type of house hold valuable articles like refrigerator, TV, computer and possession of vehicles-two and four wheeler etc., each variable was scored based on their intra variable characteristics. Per capita income was calculated by dividing the total income of the family by the number of family members. A sum of the total scores (highest and the least) was divided into four quadrants, the highest quadrant was designated as high socioeconomic (social class 1), the next in the lower orders were designated as upper-middle (social class 2), middle (social class 3) and low socio economic groups (social class 4) (Singh, Ghosh, Niaz, & Rastogi, 1997).

**Assessment of anthropometric measurements:** Each subject included for the study was measured for their body dimension and body mass: the procedures for measurements were adopted as given in Jelliffe (Jelliffe, 1966).

**Height measurement:** height was measured in centimeters using portable height measuring rod, with an accuracy of 0.1 cm.

**Weight measurement:** A battery operated digital balance (Glan electronic personal scale) was used to record the weight of boys. The balance was checked for its accuracy each time before use. Details of measurements were carried out according to WHO guideline (Group., 2006; W.H.O, 2004).

**Food frequency and dietary recall:** An interview schedule was developed to elicit information about the food intake and the quantity consumed in a day’s meal by the 24 hour recall method. The food frequency schedule was based on consumption patterns of various foodstuffs and their frequency of use.

**Dietary recall:** The 24 hour recalls method as used to obtain data about food and nutrient intake. Previously standardized cups and spoons were used as an aid to help in recalling the quantity of different foods consumed by the subjects in a 24 hour period prior to the investigation. Intakes of selected nutrients such as protein, energy, calcium, iron and β carotene were assessed. A ready reckon for nutrients, in cooked food, prepared by the department was used to compute mean intakes per day (Chachi, 2005).

**Dietary assessment:** Food frequency- this was performed in sample sizes of 6 to 10 boys per group. They were informed about the foods listed in the questionnaire and were given assistance to answer the frequency as per their household practice. As implied, each boy was given support and necessary help to complete the information.

The data were analyzed using the SPSS statistical package (version 17.5); descriptive analyses, non parametric tests (Mann-Whitney, Kruskal Wallis and Wilcoxon) were used to evaluate the data.

## 3. Results

This study included 1084 adolescent boys (6^th^–12^th^ standards) in Mysore city, south of India. Table 1 presents the family characteristics and subjective profile of the participants; it is evident that 31.2, 43.8 and 25.0 percent of boys were in pre adolescence, adolescence and post adolescence stages, respectively. Hinduism was the major religion followed by Muslims and Christians. The table also reveals the socioeconomic status of the families; 10.5% of the families were from low SES, the middle, upper, middle and higher income together formed 89.5% of the selected population. 64.7 % of the families resided in their own home. With respect to diet pattern of the families, 67.2% were non vegetarians and the rest was vegetarian.

**Table 1:** Family profile of selected adolescent boys

General profile		Total	Adolescent stages		

			Pre-adolescent 332(31.5)	Adolescent 456(43.4)	Post-adolescent 265(25.1)
**Religion**	Hindu	734(70.0)	228(68.6)	306(66.8)	201(76.0)
	Muslim	135(12.7)	46(13.8)	62(13.8)	25(9.4)
	Christian	96(9.0)	30(9.1)	55(12.0)	11(4.1)
	Others	88(8.3)	28(8.4)	33(2.7)	28(10.5)
**SES**	Low	110(10.5)	42(12.3)	42(9.2)	29(10.7)
	Middle	496(47.1)	161(48.6)	206(45.2)	128(48.4)
	Upper middle	239(22.7)	67(20.3)	119(26.2)	51(19.4)
	High	208 (19.7)	62(18.8)	89(19.8)	57(20.8)
**Type of diet**	Vegetarian	346(32.8)	104(31.3)	155(34.1)	86(32.2)
	Non- Vegetarian	707(67.2)	228(68.7)	301(65.9)	179(67.8)

Note SES: Socio Economic Status. Figures in parentheses present percentage.

The mean nutrient intake of the selected boys is presented in Table 2. It is important to mention here that, the data about nutrient intakes was computed based on 24 hours recall. The mean intake of the nutrient of the adolescent boys is presented in comparison to the RDA values suggested by ICMR for Indians (Indian Council of Medical Research, Expert group, 2009). It is obvious from Table 2 that the selected boys consumed 34.04±21.47 – 41.48±17.42 g proteins which were markedly lower than the RDA. Although the mean intake increased linearly with the advancing stages of adolescence; and the differences in intake between the groups were statistically highly significant, the intakes remained markedly lower than the RDA. Likewise, the mean intake of calories varied from 1352.06±814.959for pre-adolescent to 1539.77±697.77 for post-adolescence. The intake between the groups was 187 kcals, the differences in intake between pre-adolescence to adolescence was statistically significant while the differences in mean intakes between adolescence and post adolescence was small and was not statistically significant. Intakes of iron and β-carotene were highly variable; mean intakes along with their respective SD clearly indicate that the majority of the selected boys consumed much less than the RDAs (p<0.000), but the differences in the intakes across the stages of adolescences were not statistically significant.

**Table 2:** Mean intake of selected nutrients by the participating adolescent boys as compared to Rda

Factors			Mean Nutrient intake				

			Protein (g)	Energy (Kcal)	Calcium (mg)	Iron (mg)	β-carotene (µg)
**Adolescent stage**	Pre-adolescent	Mean±SD	34.04±21.47	1352.069±817.959	431.05±388.99	14.11±13.19	929.49±1026.68
		RDA	54.0	2190.0	600.0	34.0	700.0
		P- value^a^	<0.0001	<0.0001	<0.0001	<0.0001	<0.0001
	Adolescent	Mean±SD	40.19±21.44	1502.52±698.38	521.51±549.19	15.25±14.25	867.62±1165.62
		RDA	70.0	2450.0	600.0	41.0	1000.0
		P- value^a^	<0.0001	<0.0001	<0.0001	<0.0001	<0.0001
	Post-adolescent	Mean±SD	41.48±17.42	1539.77±697.77	540.82±370.03	15.14±11.26	824.44±994.90
		RDA	78.0	2640.0	500.0	50.0	1000.0
		P- value^a^	<0.0001	<0.0001	<0.0001	<0.0001	<0.0001
**P- value**^b^			<0.000	<0.000	<0.008	<0.124	<0.460

P value with superscript a is Wilcoxon test and with superscript b is kruskal-wallis test. *Note.* RDA: Recommended Dietary Allowance suggested by Indian Council of Medical Research.

Calcium intakes were also measured; the differences in mean intake of calcium across the adolescent stages were statistically significant. Intake of calcium by boys during pre-adolescence and adolescence stage were different by 30-40 % as compared to the RDA. However, the post-adolescent boys were found to consume a fair amount and met their RDAs. It is however interesting to note that the intake was largely different compared to the respective RDAs. (Table 2)

The adequacy of nutrient consumed by the subjects exhibited markedly lower intakes compared to the RDA (Table 3). It was therefore considered important to identify the adequacy of intakes, 70 and 80% percent of RDA (ICMR) were used as cutoff levels for comparison of the selected nutrients. Recommended Daily Allowances (RDA) include those of the U.S. Government, which recommended daily amounts for protein, vitamins and minerals for healthy adults and similarly the Indian Council of Medical Research (ICMR), New Delhi, the apex body in India for the formulation, coordination and promotion of biomedical research, is one of the oldest medical research bodies in the world. Most of the investigators have used 70% cutoff levels, on the assumption that, it would prevent severe forms of under nutrition and also that RDA envelopes a large margin of safety, therefore 70% could meet requirements for a considerable proportions of population. However, in the present study, 80% cutoff level was used because of the fact that, our subjects belong to middle to high income families. It is surprising that only a small percentage of the selected boys consumed nutrients closer to the RDA i.e., 80% cutoff level. It is evident that macro-nutrients such as calories and proteins were also consumed in significantly lower quantities and only 14% to 19 % boys were found to consume 80 % of RDA. When 70 % cutoff was employed for comparison, percent adequacy increased from 25% to 30% of the subjects (Table 3). Also of concern was that the selected boys consumed strikingly lower quantities of essential nutrients.

**Table 3:** Percent Adequacy Of Nutrients Consumed By The Adolescent Boys (Compared At 70 And 80% Cutoff Of Rda)

Stage of adolescence	% of RDA	Adequacy of Nutrients consumed				

		Protein	Energy	Calcium	Iron	B-Carotene
Pre-adolescent	70	28.3	25.9	35.0	12.9	19.2
N= 323	80	17.5	14.5	24.2	10.0	15.4
Adolescent	70	25.3	28.4	44.9	10.1	16.1
N= 456	80	13.6	19.2	35.4	7.4	14.4
Post-adolescent	70	21.5	28.8	71.1	6.6	17.7
N= 265	80	11.1	17.1	57.1	4.4	14.8

*Note.* RDA: Recommended Dietary Allowance suggested by Indian Council of Medical Research.

Table 4 suggests that estimated variance for calories, calcium and β-carotene to be very high in our study, indicating substantial inter individual variations in the intake of these nutrients. The lowest variance was found with iron and protein intakes, which indicates that the intake of boys did not differ largely and clustered around the mean. The coefficient of variation (CV) is an estimate of proportion of variation occurring in the group, i.e. the standard deviation divided by the mean expressed as a percentage. As can be seen, the CV for iron and protein was lowest (3.38 and 2.79 respectively) and a small percent of variations seemed to occur in intakes. 14% and 15% of the variations in calories and calcium intake accounted for the dispersion in intake among the selected boys. It is not surprising that the CV for β-carotene was 36% and exhibited the greater dispersion in intake among the boys. While β-carotene is the major precursor of vitamin A in Indian diets, large variations in intake is generally reported.

**Table 4:** Dispersion of intakes of nutrients by selected boys in different adolescent stages

Nutrients	Mean	SE_M_	Estimated variance	Mini	Maxi
**Protein (g)**	37.81	0.713	436.25	12.60	228.15
**Energy (Kcals)**	1409	24.960	317120.87	1000	4558
**Calcium (mg)**	479.2	12.671	107092.81	34.60	1955.62
**Iron (mg)**	14.45	0.406	110.50	2.54	68.36
**β-carotene (µg)**	83204	41.44	1168218.83	5.70	7895.48

Table 5 shows the mean intake of all the selected nutrients among the boys according to their order of birth, which indicated maximum intakes with the first born child followed by the second born. Those boys who were 3^rd^ or 4^th^ in birth order had lower intakes; however the differences were not statistically significant except for the β carotene. Socioeconomic status is a definite factor affecting the nutrient intakes; the present study also exhibits differences in the mean nutrient intakes; boys from high socioeconomic status consumed markedly higher levels of all the selected nutrients than those from low SES. The differences were not found to be statistically significant except for β carotene, Iron is not significant either. It is important to mention the problem faced while collecting data which was the resistance and indifferences exhibited by boys to answer the queries about food intake, this could be a limitation. Effect of family type on nutrient intakes showed that those boys who were 3^rd^ or 4^th^ birth order had lower intakes; however the differences were statistically significant, except for β carotene.

**Table 5:** Factors influencing nutrient intake of adolescent boys

Factors		**Mean Nutrient intake**				

		Protein (g)	Energy (Kcal)	Calcium (mg)	Iron (mg)	β-carotene (µg)
**Birth Order**	1^st^	41.845±19.78	1560±649.732	574.68±467.541	16.95±13.07	959.57±1061.22
	2^nd^	38.38±20.86	1461.367±776.922	467.381±472.38	13.75±13.44	782.3±114.51
	3^rd^	35.96±15.36	1375.78±539.776	496.179±354.145	14.39±10.03	1062.19±1363.73
	4^th^	34.41±12.32	1375.978±539.779	443.526±237.980	12.43±6.1	711.57±928.54
**p values**^a^		0.129	0.541	0.241	0.433	0.003
**SES**	Low	36.87±17.99	1399.302±620.673	465.51±339.97	13.30±9.97	653.99±803.564
	Lower Middle	37.43±20.12	1495.306±735.934	497.72±493.079	14.75±13.60	836.893±1084.34
	Upper Middle	37.53±18.19	1451.335±616.730	517.94±435.11	14.99±12.17	804.67±1103.311
	High	41.28±23.57	1543.157±801.37	548.69±464.13	16.27±14.96	1053.87±1136.85
**p values**^a^		0.000	0.000	0.000	0.124	0.404
**Family Type**	Nuclear	38.59±18.889	1451.80±667.52	495.46±405.439	15.06±12.02	899.61±1057.32
	Joint	36.81±20.83	1425.48±726.65	474.00±530.80	13.86±14.57	815.79±1062.67
	Extend	43.29±42.66	1806.87±1620.96	628.24±749.92	19.05±23.84	1307.41±1589.16
**p values**^a^		0.351	0.610	0.178	0.559	0.23
**Type of Diet**	Vegetarian	37.64±19.41	1450.098±641.414	454.79±349.055	13.84±10.3	929.06±1124.85
	Non-Vegetarian	38.44±21.64	1457.556±793.66	511.84±511.29	15.32±14.51	858.53±165.33
**p values**^b^		0.872	0.291	0.477	0.458	0.382
**Self –Esteem**	Low	38.71±29.35	1492.116±1107.669	493.71±473.59	14.36±15.68	775.43±1079.1
	Normal	36.67±18.65	1423.026±643.608	478.97±389.12	14.16±11.64	922.5±1103.0
	High	44.68±3320	1730.143±1235.752	684.58±1054.66	18.35±27.07	473.39±519.45
**p values**^a^		0.557	0.237	0.712	0.614	0.060

P value with superscript a is kruskal-wallis test and with superscript b is Mann–Whitney U test.

Table 6 provides data regarding the mean nutrient intake by the selected adolescent boys. It is obvious from the table that all the selected nutrients were consumed in varying proportions by the boys indicating frank deficiencies in their diets. Protein intake was 39.7±21.17g by boys in the normal group while the intake decreased significantly from those who were in the wasted, stunted, and wasted and stunted groups from 35.59±15.75 to 26.39±12.47 g respectively. Obese boys were found to consume the highest quantity (48.5 ±44. 56 g) of proteins. An essentially similar pattern of intake was seen with energy; compared to boys in normal group the undernourished boys consumed significantly less energy. A mean difference of 100 to 350 kcals was seen between normal, wasted, and wasted and stunted boys. The obese boys consumed highest energy with a mean difference of 230 kcals, the SD for energy intake of obese being very high and indicate a wide difference in energy intakes. Calcium also exhibited a similar pattern of intakes by the boys in different groups. Highest consumption was seen in the obese group followed by the overweight group. Calcium intake of the stunted, wasted and stunted, boys were the least accounting for 281.81±137.88 and 278.87±128.33mg per day. Iron and β carotene intakes also followed a similar trend as the other nutrients. Only the differences in mean intake of β carotene of the different groups were statistically significant. However, it is important to note that all the groups consumed considerably lower than the RDAs.

**Table 6:** Mean nutrient intake of adolescent boys according to their nutritional status

Nutritional status	Nutrient Intake				

	Protein (g)	Energy (Kcal)	Calcium (mg)	Iron (mg)	B-Carotene (µg)
**W&S**	26.3925±12.74465	1125.5688±613.68945	278.8744±150.82498	10.1900±5.01741	465.0094±609.42460
**Wasted**	35.5959±15.75977	1383.2901±620.68148	428.3697±356.02061	13.2844±9.78131	739.1226±
**Stunted**	28.4500±10.91679	1192.7800±482.35732	281.8120±137.88165	7.8240±2.98398	567.30±543.62
**Normal**	39.17±21.17205	1489.7279±732.85160	514.9054±485.67513	15.3594±14.08854	943.7823±1163.87403
**Overweight**	37.7477±16.22468	1373.3658±565.28441	532.0236±480.53744	15.0533±11.59633	965.8253±1191.22575
**Obese**	48.4905±44.56992	1720.3895±1619.94285	632.9832±697.47123	19.5282±24.51091	939.1973±1069.87606
**p values**^a^	0.225	0.196	0.348	0.151	0.011

*Note.* W&S: (wasted and stunted). P value with superscript a is kruskal-wallis test.

## 4. Discussion

Inappropriate dietary intakes during adolescence can have several sequences such as: potentially retarded physical growth, reduced intellectual capacity and delayed sexual maturation, it also affects young people’s risk for a number of immediate health problems such as iron deficiency, under-nutrition, and stunting. A deficient growth may also affect concentration, learning and school performance in school-going adolescents (Dapi, Hörnell, Janlert, Stenlund, & Larsson, 2011; Madan, Rusia, Sikka, Sharma, & Shankar, 2011). This study among Indian adolescents shows that nutrient intakes were lower than the recommendations of the ICMR which is similar to other reports (Indian Council of Medical Research, Expert, 1990). A review of the literature published from 2002 to 2009 on the nutritional status of children, aged 6 to 12 years from Latin America, Africa, Asia, and the Eastern Mediterranean region showed that the micronutrient deficiencies are a common health problem in developing countries (Srihari, Eilander, Muthayya, Kurpad, & Seshadri, 2007). Similar trends were observed in recent years among children and adolescents in India (Chakravarty, Sinha, Chakravarty, & Sinha, 2002; Haider, 2006; Rao, Balakrishna, Laxmaiah, Venekahi, & Kodali Brahman, 2006).

The present study shows that the protein intake was significantly greater among normal group boys compared to intakes among wasted, stunted, and wasted and stunted groups (39.2±21.17g vs. 35.6±15.75 to 26.4±10.91g/day, P<0.01) respectively. Obese boys were found to consume the highest quantity (48.5 ±44. 56 g) of proteins. We also observed that calcium intake of the stunted, wasted and stunted boys was significantly lower compared to obese and normal growth subjects (Tables 4, 5). Iron and β carotene intakes also followed a similar trend as the other nutrients. Our observations compared well with results reported in other studies from India (Chakravarty et al., 2002; Rao et al., 2006; Sanwalka et al., 2010). Overall, the proportion of inadequate intake was high among the adolescents in the present study. This is in accordance with other dietary studies conducted in developing countries that have reported a significant percentage of adolescents with an intake below recommendations (Dapi et al., 2011).

Adequate dietary intake of calcium during the growth period is critical for bone mineral accretion and skeletal growth. Low calcium intake during childhood and adolescence may lead to decreased bone mass accrual thereby increasing the risk of osteoporosis fractures (Chakravarty et al., 2002).

Iron deficiency is the most prevalent nutritional deficiency and the most common cause of anemia all over the world, especially in developing countries (Alton, 2005). Several studies in animals and humans have clearly demonstrated the effect of iron deficiency on development, cognition, behavior and neurophysiology (Madan et al., 2011). In the present study the majority of the selected boys consumed much less micronutrients than the RDAs. Overall, the prevalence of anemia among children from middle and high socioeconomic status ranged from 14% in the upper class in Punjab to 88% in Chennai (Srihari et al., 2007). The Punjab study revealed that more than half of the anemic children (55%) had a microcytic, hypochromic blood picture, indicating that anemia was caused by iron deficiency, other studies also support our observations (Liberona et al., 2011; Rao et al., 2006). Our results are similar to other reports who declared that 50% of adolescent children consume < 50% to 70% of the RDA (Chakravarty et al., 2002; Venkaiah, Damayanti, Nayak, & Vijayaraghavan, 2002). In light of these reports we can argue that the nutrient intakes and their adequacy observed in the present study may be closer to the actual pattern of intakes among the participant boys. Also the other eating behaviors identified in the present group like preference for snacks and missing meals frequently also support the low intakes observed in the selected groups. The situation appears grim, especially for the micronutrients; iron and β carotene intakes exhibited glaring deficiency. Calcium intake appears to be better than in some of the earlier reports which have indicated poor intake in populations (Antal et al., 2006).

Estimated variance for calories, calcium and β-carotene were very high, but for some variables the variance is estimated from severely skewed distributions. This spread of data implies that inter individual variations in intake of these nutrients to be substantial. Our results are in accordance with the other studies reported from India and other developed countries (Storey et al., 2009). Minimum and maximum intakes observed from the study support the estimated variance observed from the data.

In India, approximately 19% (190 million) of the growing population comprises school-aged children, of whom 30% (48 million) currently reside in urban India. A significant and increasing number of these children belong to middle and high socioeconomic groups. As a consequence of the socioeconomic and demographic transitions these factors are affecting developing countries such as India (Sanwalka et al., 2010). The majority of the present sample belonging to middle to high socio-economic class. Boys from high SES consumed markedly higher levels of all the selected nutrients than those from low SES. Our observations are similar to other reports (López et al., 2012; Maiti et al., 2011; Neumark-Sztainer et al., 2004; Rouhani et al., 2012; Sjöberg et al., 2003).

Those boys who were 3^rd^ or 4^th^ in birth order had lower intakes; however the differences were not statistically significant except for the β carotene. It is difficult to explain these observations; the literature also indicates an occurrence of disparity of this character (Wells et al., 2011). One of the explanations for the present observation is that the boys are from middle and high socio economic groups, since all the selected boys were from affordable families, the intake could have been essentially similar.

The nuclear family norm has become common in contemporary societies giving a different orientation to parental attitudes towards their wards. Because of smaller numbers of children in families, each child gets importance and attention from parents. In accordance with other studies, the present study also found that in nuclear and extended families the intakes of nutrient were higher than those in joint families (Zia-ud-Din, 2003). The reasons for the differences in nutrient intakes due the family type are well established and do not require further explanations. However our study has some limitations namely that the mean intakes were estimated based on one day`s food intake. It is well documented that day to day variations in nutrient intakes are quite large. However, since the observations are based on the mean of a substantial sample size from cross sectional data, it may be a fair representation for exhibiting trends of general intakes.

## 5. Conclusion

Mean intakes along with their respective SD (where appropriate) clearly indicate that the majority of the selected boys consumed protein, calories, iron, calcium and β carotene in three stages of adolescent which were markedly lower than the respective RDAs. Family type, birth order and SES correlated with nutrients intake among selected adolescent boys.
